# Gastro-Intestinal Disorders and Micronutrient Deficiencies following Oncologic Esophagectomy and Gastrectomy

**DOI:** 10.3390/cancers15143554

**Published:** 2023-07-09

**Authors:** Hugo Teixeira Farinha, Damien Bouriez, Thomas Grimaud, Ana-Maria Rotariu, Denis Collet, Styliani Mantziari, Caroline Gronnier

**Affiliations:** 1 Oeso-Gastric Surgery Unit, Department of Digestive Surgery, Magellan Center, Bordeaux University Hospital, 33600 Pessac, France; hugo.teixeira-farinha@chuv.ch (H.T.F.); damien.bouriez@chu-bordeaux.fr (D.B.); thomas.grimaud@chu-bordeaux.fr (T.G.); anamariarotariu17@gmail.com (A.-M.R.); denis.collet@chu-bordeaux.fr (D.C.); 2 Department of Visceral Surgery, University Hospital of Lausanne, Rue du Bugnon 46, 1011 Lausanne, Switzerland; styliani.mantziari@chuv.ch; 3 Faculty of Biology and Medicine, University of Lausanne (UNIL), 1015 Lausanne, Switzerland; 4 Faculty of Medicine, Bordeaux Ségalen University, 33000 Bordeaux, France

**Keywords:** micronutrient deficiencies, esophagectomy, gastrectomy, malnutrition

## Abstract

**Simple Summary:**

Despite considerable advances in esogastric cancer surgeries, postoperative malnutrition remains a significant yet overlooked challenge. It triggers weight loss, muscle mass reduction, and essential nutrient deficiencies, detrimentally impacting patients’ quality of life and prognosis. Our study reveals that micronutrient deficiencies are just as prevalent in patients post-esophagectomy as after partial or total gastrectomy. These findings underscore the need for proactive measures, including prevention, early detection, and prompt management.

**Abstract:**

Primary surgical indications for the esophagus and stomach mainly involve cancer surgeries. In recent years, significant progress has been made in the field of esogastric surgery, driven by advancements in surgical techniques and improvements in perioperative care. The rate of resectability has increased, and surgical strategies have evolved to encompass a broader patient population. However, despite a reduction in postoperative mortality and morbidity, malnutrition remains a significant challenge after surgery, leading to weight loss, muscle mass reduction, and deficiencies in essential nutrients due to digestive complications. Malnutrition worsens quality of life and increases the risk of tumor recurrence, significantly affecting prognosis. Nevertheless, the nutritional consequences following surgery are frequently overlooked, mainly due to a lack of awareness regarding their long-term effects on patients who have undergone digestive surgery, extending beyond six months. Micronutrient deficiencies are frequently observed following both partial and total gastrectomy, as anticipated. Surprisingly, these deficiencies appear to be similarly prevalent in patients who have undergone esophagectomy with iron, vitamins A, B1, B12, D, and E deficiencies commonly observed in up to 78.3% of the patients. Recognizing the distinct consequences associated with each type of intervention underscores the importance of implementing preventive measures, early detection, and prompt management.

## 1. Introduction

The ongoing progress in oncological esogastric surgery has led to increased patient survival rates and improved quality of life [1,2]. However, these surgeries can have detrimental effects on patients’ digestive and functional abilities [3,4], highlighting the importance of considering the nutritional implications for long-term outcomes [5].

Malnutrition prevalence varies across different tumor sites, with rates ranging from 80–85% in pancreatic cancer, 65–75% in head and neck cancer, 45–60% in lung cancer, and 30–60% in colorectal cancer [6]. Prior to major surgery, approximately 80% of patients with esogastric cancer experience significant weight loss, and about 27% are already malnourished [7,8,9]. Several indices, such as significant weight loss (>10% of basal body weight), low body mass index (BMI) (<18 kg/m²), and reduced levels of albumin (<30 g/L) and pre-albumin (<0.20 mg/dL), can help the diagnosis of malnutrition [10]. Elderly patients and those with sarcopenia (i.e., low muscle quantity and quality) require special attention, as they are particularly vulnerable to malnutrition [11]. Furthermore, it should be noted that even obese patients can experience malnutrition, especially in the context of sarcopenic obesity [11]. A grading system based on BMI and percent weight loss (%WL) has shown significant survival differences among cancer patients [12]: patients with higher %WL and lower BMI categories have notably shorter median survival compared to other grades.

Malnutrition can arise from various mechanisms that impact both the local and systemic aspects of the disease. Locally, the tumor itself can cause symptoms such as odynophagia (painful swallowing) and dysphagia (difficulty swallowing), resulting in reduced nutrient intake. Additionally, tumors in the upper gastrointestinal tract can hinder gastric emptying, further exacerbating malnutrition [13]. Systemically, cancer can induce cachexia, through the release of pro-inflammatory cytokines and chronic inflammation, leading to muscle wasting, weight loss, and metabolic alterations [14]. Moreover, neoadjuvant treatments, such as chemotherapy and radiation therapy, can have anorexigenic effects and cause side effects like esophagitis, mucositis, and malabsorption, which further compromise nutritional status [15]. Psychosocial factors, including fear, anxiety, and depression, can also negatively impact appetite and food intake [16]. Understanding these mechanisms is crucial for implementing appropriate nutritional interventions to address and mitigate malnutrition in cancer patients.

Malnutrition also impacts neoadjuvant treatment, increasing therapy intolerance and medication toxicity. Reductions of up to 10% in chemotherapy doses have been observed due to malnutrition [17]. Additionally, malnutrition significantly impairs patients’ quality of life, causing fatigue and general debilitation, according to the World Health Organization criteria [18]. Furthermore, surgical procedures in malnourished patients compromise their ability to handle physiological stress, including a weakened immunological response [19].

Micronutrient deficiencies are prevalent after esogastric surgery [5], as anatomical and functional modifications resulting from surgical resection and reconstruction directly impact their absorption [13]. Despite the significance of these consequences, micronutrient deficiencies are often underdiagnosed or undertreated in patients who have undergone oncological esogastric surgery [5]. It is critical to prioritize the prevention and treatment of micronutrient deficiencies, as they can significantly improve long-term outcomes for these patients [5].

This article provides a comprehensive review on malnutrition mechanisms and micronutrient deficiencies and their consequences after esophagectomy or gastrectomy for cancer.

## 2. Materials and Methods

We conducted a literature search of the PubMed, MEDLINE and EMBASE databases, which was conducted by four authors (H.T.F., T.G., A.-M.R. and C.G.), to identify the literature related to micronutrient deficiencies following esophagectomy and gastrectomy. Search terms included controlled terms from the MeSH database in PubMed and MEDLINE and the EMtree in the EMBASE database, as well as free text terms. Terms expressing “esophageal cancer”, “esophagectomy”, “gastric cancer”, “gastrectomy”, “malnutrition”, “micronutrient deficiency”, “malabsorption” and “metabolic complications” in all databases, were variously combined in the search.

## 3. The Mechanisms Involved in Malnutrition following Esophagectomy

Esophagectomy is the gold standard for treating resectable esophageal cancer. Perioperative and intraoperative advancements, particularly the minimally invasive approach, along with the progress in anesthesia, have significantly reduced postoperative mortality and morbidity [1]. In recent years, there has been a growing focus on assessing postoperative long-term quality of life as an important measure of success. Different surgical procedures are employed based on tumor location, extent, and overall health status. Lewis–Santy esophagectomy with a minimally invasive thoracoabdominal approach is preferred for tumors in the middle-lower third of the esophagus [20]. For tumors in the upper third, the McKeown esophagectomy or cervical thoracoabdominal approach is preferable [21]. In cases of tumors in the lower third, a transhiatal esophagectomy may be considered, especially for patients with respiratory frailty, avoiding a thoracic approach which can impair the respiratory function [22]. The use of the stomach as a substitute for the esophagus is the standard situation in these surgeries, and can lead to anatomical and physiological changes, resulting in various clinical symptoms. The severity of these symptoms, such as abnormal thoracic stomach motility, delayed gastric emptying, dumping syndrome, and reflux, depends on the shape, size, pathway, and position of the remodeled stomach in the thorax [23]. These changes have implications for postoperative nutrition [23]. Consequently, malnutrition is a common complication following esophagectomy, resulting from decreased intake, malabsorption, and altered metabolism. Thus, patients may experience weight loss, difficulty eating, and nutrient deficiencies [24]. At one year post-esophagectomy, 55% of patients lost more than 10% of their usual weight, doubling their risk of tumor recurrence, with malnutrition being a significant factor affecting prognosis [25]. Therefore, meticulous nutritional evaluation and management is crucial in the postoperative period to ensure optimal recovery and quality of life for patients.

### 3.1. Gastric Motility Disorders

Gastric motility disorders can manifest as either accelerated or delayed gastric emptying [26]. Gastroplasty, due to various mechanisms such as reduced gastric capacity, disturbed inhibitory entero-gastric hormonal reflexes, and decreased gastric vascularization, can lead to an acceleration of initial gastric emptying [27] often associated with motor diarrhea [28]. Delayed gastric emptying is the most frequently observed issue in patients with motility dysfunction of the thoracic stomach, with an incidence of 50% following esophagectomy [26]. It can result in symptoms like early satiety, postprandial heaviness, regurgitation, and dysphagia [28]. Reduced stomach volume weakens receptive expansion, and degeneration of the myenteric plexus caused by gastric tubulization and vagotomy contributes to delayed emptying [29]. A study suggests that the myenteric plexus and the remaining vagus nerve in the antrum of the lesser curvature can restore contractility in the thoracic stomach [30]. A 2–4 cm narrow gastric tube after Ivor Lewis was proposed to improve postoperative quality of life for esophageal cancer patients without increasing the risk of complications [31]. Procedures such as endoscopic myotomy (POEM), pyloroplasty or pacemaker implementation have been proposed as treatments for delayed gastric emptying, but their use after esophagectomy remains controversial due to the potential risks of fistula, bile reflux, and dumping syndrome [32] [33]. Medications like erythromycin used as a prokinetic can be administered to treat delayed gastric emptying, by activating motilin receptors in the smooth muscles of the gastric antrum and duodenum [34].

### 3.2. Gastroesophageal or Biliopancreatic Reflux Disease (GERD)

More than 60% of patients report gastroesophageal or biliopancreatic reflux after esophagectomy, which justifies preventive treatment with preprandial proton pump inhibitors for life, 30 min before a meal [35]. Gastroplasty reconstruction and intra-thoracic anastomosis abolishes the anti-reflux mechanism related to the esophageal hiatus [23]. Various factors can aggravate reflux, such as delayed gastric emptying, gastric denervation, and the negative intrathoracic pressure promoting reflux. Reflux can lead to clinical manifestations including bile and gastric acid-induced laryngitis, vomiting, repeated coughing, pneumonia, and inability to lie in a supine position [36]. To reduce the discomfort associated with reflux, patients should follow hygiene-dietary guidelines, such as dividing meals, waiting 2 h post-meal before lying down, and potentially sleeping with multiple pillows [36].

### 3.3. Dysphagia

Primarily associated with the development of anastomotic stricture, this complication typically arises during the initial months following surgery. Benign strictures commonly observed are peptic strictures, occurring with estimated frequencies of 10–20% for cervical anastomosis and 25% for intrathoracic anastomosis [37]. In cases involving coloplasty, dysphagia is more frequently attributed to the occurrence of an anastomotic stricture phenomenon, which is associated with vascularization disorders of the implant and can affect approximately 30–45% of patients [38].

### 3.4. Dumping Syndrome

Dumping syndrome is a common complication caused by abnormal motility of the stomach located in the thorax, resulting in rapid arrival of partially digested rich or sugary foods into the duodenum, which leads to a discomfort characterized by nausea and vomiting. Five percent of patients show moderate symptoms and one percent show severe symptoms [23,26]. Eating habit modifications, such as having multiple small meals, avoiding monosaccharides, and increasing fat and protein intake, can alleviate symptoms. In severe cases, drugs such as propranolol can be administered [39].

### 3.5. Diarrhea

Diarrhea following esophagectomy can be attributed to several mechanisms related to the disruption of the normal digestive process. Surgical procedures alter the anatomy and function of the gastrointestinal tract, impacting its motility [40]. One significant contributing factor is osmotic diarrhea caused by lactose intolerance. After surgery, the rapid clearance of lactose-containing products can overload the lactose-hydrolyzing capacity, leading to osmotic diarrhea in adults [40]. Additionally, diarrhea may be associated with a multifactorial malabsorption syndrome, including exocrine pancreatic insufficiency (EPI), small intestinal bacterial overgrowth (SIBO), and bile acid malabsorption (BAM) [40]. EPI occurs when the pancreas fails to produce sufficient digestive enzymes for optimal nutrient absorption. Incidence rates for EPI range from 16% to 100%, while SIBO ranges from 37.8% to 100%, and BAM from 4% to 100% [40]. The development of these syndromes can vary, with EPI occurring between 21 days and 60 months post-esophagectomy, SIBO within 1 to 24 months, and BAM within 1 to 24 months. Treatment modalities such as pancreatic enzyme replacement therapy, rifaximin, and colesevelam have shown promising results in improving symptoms and weight management in related studies [40,41].

## 4. Micronutrient Deficiency following Esophagectomy

Previous studies in bariatric and gastric cancer surgery have shown that micronutrient deficiencies can worsen or develop in a substantial number of patients [42]. Iron and vitamin A, B1, B12, D, and E deficiencies are commonly observed [24]. Four studies that investigated micronutrient deficiencies in patients following an esophagectomy were identified [24,40,43,44].

Approximately 18% of patients undergoing esophagectomy with gastric tube reconstruction for cancer are likely to experience vitamin B12 deficiency, according to Van Hagen [43]. In this cross-sectional study, 11/99 patients (11%) were found to have vitamin B12 deficiency, with a median duration of 19.3 months between surgery and 5/88 patients (5.6%) of a prospective cohort had preoperative deficiency, and an additional 10.2% developed deficiency at a median time of 6 months after the operation. The estimated one-year incidence of vitamin B12 deficiency was 18.2%.

Heneghan et al. [40] investigated disease-free patients who underwent esophageal or gastric oncologic resections with a minimum follow-up of 18 months. There were no notable alterations in corrected calcium, phosphate, magnesium, vitamin E, and vitamin D, but a significant decline in vitamin A levels was observed at 1 year (*p* = 0.005) and 2 years (*p* = 0.017) after surgery compared to baseline, indicating a potential issue with fat absorption. This decrease in vitamin A levels suggests a possible impairment in its absorption [40].

In a retrospective cohort study including 75 patients operated on for esophageal cancer without lymph node involvement [44], Elliot et al. found that 25% were diagnosed with osteoporosis, with bone mineral density declining significantly at the first and second year postoperatively (*p* < 0.0001). A deficiency in vitamin D of 21% was observed in immediate preoperative patients, reaching as high as 26% at one year, and 11% at two years postoperatively. Concurrently, the prevalence of osteoporosis rose to 38% and 44% in the first and second year, respectively (*p* = 0.049). This paper concluded that osteoporosis is common in this patient population, emphasizing the need for strategies that minimize bone mineral density decline to mitigate the risk of fragility fractures [44].

In a recent study that evaluated the micronutrient levels of 83 patients who underwent minimally invasive esophagectomy for cancer [24], it was found that, after a median duration of 6.1 months, 78.3% of the patients exhibited at least one deficiency in micronutrients. Furthermore, 37.3% of the patients had multiple deficiencies, which included iron, vitamin B12, and vitamin D. Folate deficiency and anemia were also significant. After 24.8 months, deficiencies persisted but were mostly rectified with supplementation. The research suggests regular monitoring and supplementation for such patients, as deficiencies can persist for up to 24 months. Iron deficiency was most prevalent (31.3%), followed by folate (23.5%) and vitamin D (11.8%). Anemia was found in three patients; two with abnormal MCV levels [24].

## 5. The Mechanisms Involved in Malnutrition following Gastrectomy

Gastrectomy, which involves the surgical removal of all or part of the stomach, has become the preferred treatment for malignant gastric lesions [2]. Partial and total gastrectomy is often performed along with D2 lymph node dissection according to current international guidelines [45,46,47]. The gold standard for post-partial gastrectomy reconstruction is the gastrojejunostomy with a Roux-en-Y anastomosis, utilizing a jejunal loop in a transmesocolic or ante mesocolic configuration [48]. This type of reconstruction can lead to malabsorption similar to that seen in gastric bypass, regardless of the length of the intestinal loops involved during Roux-en-Y reconstruction. The malabsorption can be explained by a reduction in nutrient absorption, changes in gastric acid production, alterations in diet, or decreased appetite and food intake [12]. The extent of alteration of oral intake will be determined by the size of the gastric remnant. Total gastrectomy results in significant weight loss, averaging 15% of preoperative weight [49]. Over half of these patients fail to regain their pre-surgical weight [50]. On the contrary, after partial gastrectomy, weight loss is moderate, and approximately more than 80% patients return to a healthy weight [51].

### 5.1. Roux-en-Y Limb Syndrome

After Roux-en-Y reconstruction, approximately one-third of patients experience early satiety, which is linked to disturbed gastric emptying caused by jejunal transection, resulting in decreased peristalsis and gastric stasis, especially in distal gastrectomy cases [52]. This motor dysfunction of the constructed jejunal limb is independent of vagotomy [53]. The syndrome presents with abdominal distension, nausea, and vomiting, due to altered anatomy and changes in gastrointestinal transit following the procedure. Food stasis in the by-passed stomach and Roux limb can lead to discomfort, while the rapid transit of food through the small intestine can contribute to malabsorption and the risk of malnutrition. Hormonal alterations in appetite regulation and nutrient absorption further influence the syndrome. Management involves dietary modifications, nutritional supplementation, and careful monitoring, to prevent long-term complications associated with malnutrition [54].

### 5.2. Small Gastric Remnant Syndrome

Small gastric remnant syndrome (or early satiety syndrome) is a condition that arises when the stomach loses its reservoir function, typically when over 80% of the stomach has been removed. This situation often leads to symptoms like premature fullness, discomfort in the upper abdomen after eating, and vomiting. It is not uncommon for individuals to experience weight loss and deficiencies in vitamins and minerals, akin to other conditions that follow stomach surgery. The main treatment approach involves dietary changes [52].

### 5.3. Modification of Ghrelin Synthesis

The collapse of ghrelin is the main cause of anorexia post-gastrectomy [55]. Approximately 70% of ghrelin peptide is produced by gastric parietal cells, and after being synthesized, it enters the bloodstream before playing a significant role as a hormone that stimulates appetite. One-third of patients show no recovery of appetite as it was before surgery and the main underlying mechanisms are multifactorial. Meanwhile, some research suggests ghrelin replacement could help alleviate these complications by restoring appetite and promoting weight gain [55].

### 5.4. Gastroparesis and Dumping Syndrome

Gastroparesis, leading to symptoms like early satiety, feeling full after meals, and nausea or vomiting, is often under-diagnosed, and it can occur in 5% to 25% of patients after partial gastrectomy, particularly affecting elderly patients and women [56]. Dumping syndrome is a frequent complication that can occur post-surgery, typically after vagotomy procedures. This syndrome is characterized by a quick movement of food from the stomach to the small intestine, leading to early gastrointestinal symptoms and later hypoglycemia. The incidence of dumping syndrome after surgery can be as high as 30% [4].

## 6. Micronutrient Deficiency following Gastrectomy

### 6.1. Iron Deficiency

The prevalence of iron deficiency at two years after gastrectomy was observed between 40 and 70%, and iron deficiency anemia was seen in 31% of patients [57]. Iron deficiency anemia is a prevalent sequela following partial or total gastrectomy. The duodenum and the upper part of the small intestine, primary sites for iron absorption, are typically bypassed during most gastrectomy procedures, thus impeding the efficient uptake of iron. Furthermore, the expedited transit of food through the intestine, as a result of the surgery, restricts the time available for iron absorption [58]. Concomitantly, partial or total gastrectomy results in diminished gastric acid availability in the small intestine; a crucial component that facilitates the conversion of iron into a form that is more bioavailable. Collectively, these physiological alterations, coupled with a probable diminished consumption of iron-rich foods, contribute to the incidence of iron deficiency post-gastrectomy [58]. Treatment of iron deficiency depends on its severity and involves oral or parenteral administration of elemental iron. However, oral supplementation does not always normalize ferritin levels, and intravenous supplementation has proven more effective [57,58].

### 6.2. Copper and Zinc Deficiency

Roux-en-Y procedures constitute the primary source of acquired copper deficiency, responsible for approximately 50% of documented instances [59]. Copper, primarily absorbed within the stomach and proximal segment of the duodenum, plays a critical role in hematopoiesis and neurologic system function. Deficiency of this essential micronutrient can lead to manifestations such as microcytic anemia, neutropenia, and ataxia, which can be further exacerbated with the administration of iron supplementation [60]. Zinc absorption also occurs within the duodenum and jejunum, leading to the proposition that patients’ may experience altered absorption dynamics post-gastrectomy. Nevertheless, limited studies have ventured into examining the relationship between clinicopathological characteristics and serum zinc concentrations. The prevalence of zinc deficiency ranges from 10 to 75% in patients following a gastrectomy procedure and, of course, gastric bypass [59].

### 6.3. Calcium and Phosphor Deficiency

Several mechanisms contribute to the development of calcium deficiency, including insufficient dietary intake, duodenal bypass which is the primary site of calcium absorption, decreased dissolution of calcium salts due to hypochlorhydria, and vitamin D deficiency [61]. Calcium deficit is one of the main causes of bone disorders following gastrectomy [62]. The scientific literature also suggests a likely role of gastrocalcin, a hormone produced by enterochromaffin-like (ECL) cells in response to gastrin [63]. Consequently, gastrin indirectly affects bone health by triggering the release of gastrocalcin, which in turn would induce osteoclast activation. Five years post-gastrectomy, over three-quarters of patients have diminished phosphatemia levels compared to the serum levels of control subjects [61]. The causes are primarily linked to insufficient intake, bypass of the gastric absorption site, and vitamin D deficiency.

### 6.4. Fat-Soluble Vitamins

The jejunum and ileum are identified as the key absorption sites for fat-soluble vitamins, which includes vitamins A, D, E, and K [5,64]. Undergoing surgical procedures such as Roux-en-Y can significantly heighten the risk of developing deficiencies in these vitamins. Vitamin A deficiency can manifest as visual disorders like night blindness and xerophthalmia, as well as skin conditions such as keratomalacia and follicular hyperkeratosis. Deficiency of vitamin D, on the other hand, can contribute to skeletal deformities including rickets and osteomalacia. Vitamin E deficiency may cause neurological issues like sensory and motor neuropathy, and ataxia, alongside retinal degeneration and hemolytic anemia. Vitamin K deficiency is primarily associated with hemorrhagic disease, a severe clotting disorder [64].

### 6.5. Vitamin D Deficiency

After a total gastrectomy, there is an increased risk of lowered bone mineral density, possibly due to vitamin D deficiency. While research findings remain uncertain, they generally suggest that the levels of vitamin D may decrease after surgery up to 36% [5]. Bone density seems to decrease post-gastrectomy, with the type of surgery impacting vitamin D levels. While vitamin D supplementation is not standardized post-gastrectomy, it has been shown to improve bone density, according to reviewed studies [5,65].

### 6.6. Vitamin B1 Deficiency

Vitamin B1 (thiamine) deficiency, primarily absorbed in the duodenum and proximal jejunum, can occur within three weeks post-surgery in patients with persistent vomiting or severely reduced oral intake [43]. Asymptomatic, low thiamine concentrations have been reported in 1 to 49 percent of post-Roux-en-Y patients, depending on the type of surgery and length of follow-up. The most frequent manifestation of thiamine deficiency post-surgery is Wernicke encephalopathy [65].

### 6.7. Vitamin B12 Deficiency

Postoperative prevalence is notably higher after Roux-en-Y total gastrectomy, with about one-third or more patients affected [43]. This deficiency arises due to insufficient gastric acid, needed for cleaving vitamin B12 from dietary protein, and potentially diminished intrinsic factor. This deficiency, causing permanent megaloblastic anemia, requires lifelong management via oral or parenteral supplements [43]. B12 deficiency is typically detected two years or more after surgery due the 12-to-18-month B12 storage capacity of the body, hence the need for annual long-term laboratory monitoring [5,43,66].

### 6.8. Folate Deficiency

Folate deficiency, while less common than vitamin B12 deficiency due to its absorption across the entire small intestine, can also lead to megaloblastic anemia. Postoperative malabsorption of folate is less common following Roux-en-Y [5,24].

## 7. Management of Postoperative Malnutrition

### 7.1. Postoperative Nutrition

Enhanced recovery after surgery (ERAS) protocols have been successfully introduced in patients undergoing an esophagectomy. Nonetheless, the initiation of oral intake remains an area of ongoing debate [67]. Comparative studies have demonstrated enteral nutrition’s association with a decrease in postoperative complications and shorter durations of hospital stay, relative to parenteral nutrition [68]. Early enteral nutrition is supported by strong evidence to decrease the prevalence of severe complications following esophagectomy [67]. Nutritional therapy can be achieved safely via enteral nutrition as demonstrated by the NUTRIENT II trial or parenteral nutrition. Unfortunately, some patients cannot achieve early oral nutrition after esophagectomy, most of the time due to postoperative complications [69]. Despite this, enteral is always the preferred nutritional therapy route after esophagectomy, which can also be administered via jejunostomy or nasoenteral tube [70]. Compared to a nasogastric tube, jejunostomy has demonstrated superior results in terms of postoperative pneumonia rates, length of hospital stay, and catheter dislocation. Moreover, the surgical placement of a feeding jejunostomy, which can be easily performed via laparoscopy, has been associated with low mortality rates (0–0.5%) and re-operation rates (0–2.9%). Between postoperative days 3 and 6, patients with a jejunostomy met 88 to 100% of their nutritional requirements [71,72].

Regarding partial or total gastrectomy, guidelines strongly suggest the introduction of prompt postoperative nutrition in order to decrease the catabolic effects facilitating quicker intestinal function restoration, in order to minimize potential complications and decrease the duration of hospitalization [73,74]. Tailored nutritional support is suggested for patients who cannot meet at least 60% of their necessary caloric consumption [73]. It recommends prioritizing high-energy oral feeds, with enteral tube feeding as an option when oral intake is not possible, and parenteral nutrition only when the gut is dysfunctional or unreachable [73]. Feeding tube placement is common among patients undergoing gastrectomy. Specific complications related to jejunostomy are between 3.1 and 12%, according to a published series [75]. Current guidelines recommend consideration of a feeding jejunostomy tube for all patients undergoing resection for gastric cancer [74].

### 7.2. Micronutrient Monitoring

The existing literature presents no conclusive data regarding micronutrient deficiencies following esophagectomy [24]. However, recent studies highlighted that the vast majority of patients had at least one micronutrient deficiency six months post-operation, including deficiencies in vitamin B12, vitamin D, folate, and iron [24,40,43,44]. Similar to bariatric surgery, esophagectomy patients may develop micronutrient deficiencies due to anatomical changes that lead to adverse gastrointestinal symptoms. Contrary to practices in bariatric surgery and after total gastrectomy with Roux-en-Y, no universally accepted protocol exists for tracking and replacing micronutrients in patients who have had an esophagectomy. Given the potential nutritional deficiencies inherent to esophagectomy or partial gastrectomy, it would be beneficial to have a multidisciplinary team comprising a nutritionist to supervise comprehensive nutritional balance and assess vitamin levels at intervals of 1 month, 3 months, 6 months, 1 year, 2 years, and up to 5 years. This would allow for accurate identification of the emergence of deficiencies.

## 8. Conclusions

In conclusion, micronutrient deficiencies are common following esophagectomy or partial and total gastrectomy for cancer treatment. The onset of micronutrient deficiencies typically manifests between 6 and 24 months postoperative. By raising awareness of these issues, we aim to encourage healthcare providers to prioritize the prevention and treatment of micronutrient deficiencies in patients who have undergone oncological esogastric surgery. Regular checks are suggested for these patients, and if deficiencies are found, they should be supplied with the needed micronutrients to avoid long-term complications, which include neurological disorders, bone disease, or anemia. In addition, other factors, such as preoperative malnutrition, early and late postoperative complications, and the employment of neoadjuvant and adjuvant treatments, should also be taken into consideration due to their potential to intensify micronutrient deficiencies.
